# Supplementary material for: Identification of photoperiod-induced specific miRNAs in the adrenal glands of Sunite sheep (Ovis aries)
Source: Front Vet Sci. 2022 Jul 22;9:888207. doi: 10.3389/fvets.2022.888207 (PMC9354845; doi:10.3389/fvets.2022.888207)
Supplement: Supplementary file 1 [file Data_Sheet_1.ZIP › Supplementary file/miRDeep2 file.zip!/miRDeep2 file/Know_Structure/LP42A1/oar-mir-154a.pdf]

miRBase precursor : oar-mir-154a
Total read count : 449
oar-miR-154a-5p read count : 4
oar-miR-154a-3p read count : 445
remaining reads : 0

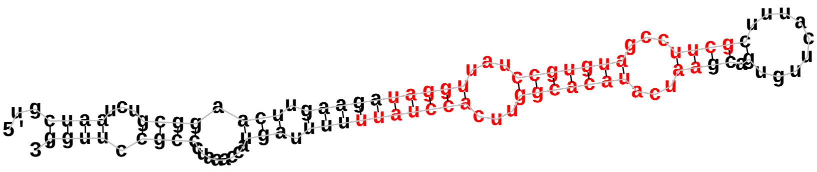

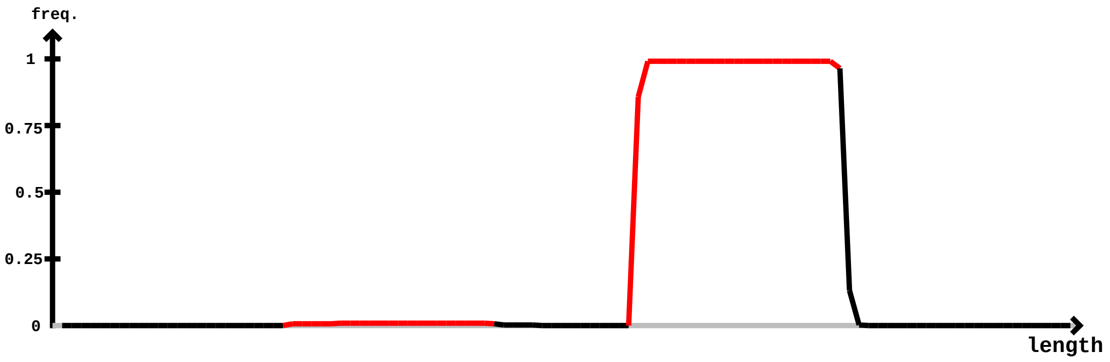


```
                                oar-miR-154a-5p
                                                  oar-miR-154a-3p
5'- ugcuaaucugcggaacuugaagauagguuauccguguagccuucgcuuuacuugugacgaaucauacacgguucaccuauuuuuuaguaccaaaucccgccuugg   -3'    exp
    .((......))((.(((.((((((((((...(((((((...(((((.........).))))...)))))))...)))))))))).))).)).............    reads     mm        sample
    .......................uagguuauccguguagccuuc.............................................................    1         0         seq
    .......................uagguuauccguguagccuucg............................................................    2         0         seq
    ............................uauccguguagccuucgcuuu........................................................    1         0         seq
    ...........................................................aaucauacacgguucaccuau.........................    12        0         seq
    ...........................................................aaucauacacgguucaccuauu........................    361       0         seq
    ...........................................................aaucauacacgguucaccuauuu.......................    12        0         seq
    ............................................................aucauacacgguucaccuauu........................    13        0         seq
    ............................................................aucauacacgguucaccuauuu.......................    46        0         seq
    ............................................................aucauacacgguucaccuauuuu......................    1         0         seq
```
